# Electrophysiological Method for Whole-cell Voltage Clamp Recordings from *Drosophila* Photoreceptors

**DOI:** 10.3791/55627

**Published:** 2017-06-13

**Authors:** Ben Katz, Rita Gutorov, Elisheva Rhodes-Mordov, Roger C. Hardie, Baruch Minke

**Affiliations:** ^1^Department of Medical Neurobiology, Faculty of Medicine and the Edmond and Lily Safra Center for Brain Sciences (ELSC), Hebrew University; ^2^Department of Physiology, Development and Neuroscience, University of Cambridge

**Keywords:** Neuroscience, Issue 124, Whole-cell recordings, voltage clamp, current clamp, electrophysiology, *Drosophila melanogaster*, phototransduction, single-cell Ca^2+^ imaging, intracellular perfusion

## Abstract

Whole-cell voltage clamp recordings from *Drosophila melanogaster* photoreceptors have revolutionized the field of invertebrate visual transduction, enabling the use of *D. melanogaster* molecular genetics to study inositol-lipid signaling and Transient Receptor Potential (TRP) channels at the single-molecule level. A handful of labs have mastered this powerful technique, which enables the analysis of the physiological responses to light under highly controlled conditions. This technique allows control over the intracellular and extracellular media; the membrane voltage; and the fast application of pharmacological compounds, such as a variety of ionic or pH indicators, to the intra- and extracellular media. With an exceptionally high signal-to-noise ratio, this method enables the measurement of dark spontaneous and light-induced unitary currents (*i.e. *spontaneous and quantum bumps) and macroscopic Light-induced Currents (LIC) from single *D. melanogaster* photoreceptors. This protocol outlines, in great detail, all the key steps necessary to perform this technique, which includes both electrophysiological and optical recordings. The fly retina dissection procedure for the attainment of intact and viable *ex vivo* isolated ommatidia in the bath chamber is described. The equipment needed to perform whole-cell and fluorescence imaging measurements are also detailed. Finally, the pitfalls in using this delicate preparation during extended experiments are explained.

**Figure:**
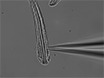


## Introduction

Extensive genetic studies of the fruit fly, *Drosophila melanogaster (D. melanogaster)*, initiated more than 100 years ago, have established the *D. melanogaster *fly as an extremely useful experimental model for the genetic dissection of complex biological processes. The methodology described below combines the accumulated power of *D. melanogaster* molecular genetics with the high signal-to-noise ratio of whole-cell patch clamp recordings. This combination allows for the study of *D. melanogaster* phototransduction as a model of inositol-lipid signaling and TRP channel regulation and activation, both in the native environment and at the highest resolution of single molecules.

Application of the whole-cell recording method to *D. melanogaster* photoreceptors has revolutionized the study of invertebrate phototransduction. This method was developed by Hardie^1^ and independently by Ranganathan and colleagues^2^ ~26 years ago and was designed to exploit the extensive genetic manipulation tools of *D. melanogaster *and use them to uncover mechanisms of phototransduction and inositol-lipid signaling. At first, this technique suffered from a rapid reduction in light sensitivity and a low yield of ommatidia during the dissection process, which prevented detailed quantitative studies. Later, the addition of ATP and NAD to the patch pipette dramatically increased the suitability of the preparation for prolonged quantitative recordings. Thereafter, extensive characterization of the signal-transduction mechanism at the molecular level was realized.

Currently, *D. melanogaster* phototransduction is one of the few systems in which phosphoinositide signaling and TRP channels can be studied *ex vivo* at single-molecule resolution. This makes *D. melanogaster* phototransduction and the methodology developed to study this mechanism a highly sensitive model system. This protocol describes how to dissect the *D. melanogaster* retina and mechanically strip the isolated ommatidia from the surrounding pigment (glia) cells. This enables the formation of a giga-seal and a whole-cell patch clamp on the photoreceptor cell bodies. Fortunately, most signaling proteins are confined to the rhabdomere and do not diffuse. In addition, there is an immobile Ca^2+^ buffer called calphotin, located between the signaling compartment and the cell body^3^,^4^, and a high expression level of the Na^+^/Ca^2+^ exchanger (CalX) in the microvilli^5^. Together, the protein confinement to the rhabdomere, the calphotin buffer, and the high expression of the CalX allow for relatively prolonged (*i.e. *up to ~20 min) whole-cell recordings, without the loss of essential components of the phototransduction process and while maintaining high sensitivity to light. The following protocol describes how to obtain isolated ommatidia and perform whole-cell recordings that appear to preserve the native properties of the phototransduction cascade. Whole-cell patch clamp experiments on dissociated cockroach (*Periplaneta americana*)^6^ and cricket (*Gryllus bimaculatus*)^7^ ommatidia were performed similarly to that described for *D. melanogaster*. In addition, patch clamp experiments on dissociated photoreceptors of the file clam, (*Lima scabra*) and scallop (*Pecten irradians*) were performed in a slightly different manner from that conducted on *D. melanogaster*, allowing both whole-cell^8^ and single-channel measurements^9^. Here, the major achievements obtained in *D. melanogaster* using this technique are described. The Discussion includes the description of some pitfalls and limitations of this technique.

## Protocol

### 1. Reagent Preparation

NOTE: Prepare all solutions according to the instructions in **Tables 1-4**.

1. Fill a 10 mL syringe with Extracellular Solution (ES or ES-0Ca^2+^; as required; see **Table 1**) and keep it on ice.
2. Prepare one vial of Trituration Solution (TS; see **Table 2**; *i.e.* ES or ES-0Ca^2+^ + FBS and sucrose) and keep this on ice .
3. Prepare sufficient ES for the experiment and keep this on ice until needed. NOTE: Continuous bath perfusion is not required, so the volume of ES need not be more than a few dozen mL.
4. Using a 22 µm PVDF filter, load the intracellular solution (see **Table 3 **or** Table 4**) into a 1 mL syringe with an electrode filling elongated tip. Keep this on ice.

### 2. General Setup of Dissecting Tools


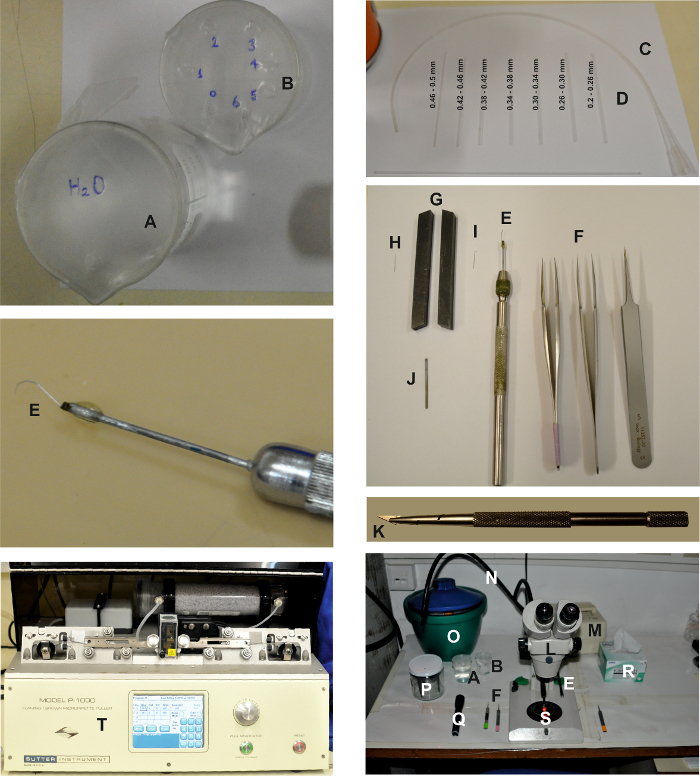
**Figure 1: Tools and Devices Needed for Making the Isolated Ommatidia Preparation.** The pictures show the various devices required for creating the isolated ommatidia preparation, as described in the detailed protocol above. Two beakers, one filled with water*** (*****A**) and the other filled with ethanol (**B**) for cleaning the trituration pipettes (**D**), which are connected to tubing (**C**). The dissecting tools are: 2 pairs of fine and 1 pair of coarse tweezers (**F**) and a retina scooper (**E**). In order to prepare the retina scooper, a micro dissection needle (**H**) is pressed between two lathe tools acting as a vice (**G**) to flatten the top of the needle (**I**). Then it is connected to an elongated piece of metal using glue (**J**) and mounted onto a needle holder (**E**). Razor blade chip and holder: Break off and mount a small triangular chip of a razor blade using a razor blade holder (**K**). The dissection working area is composed of a binocular (**L**) and a cool red light source ((**M),**) with two light guides (**N**). The dissection tools, including tweezers (**F**), the retina scooper (**E**), beakers (**A and B**), a flashlight with a red filter (**Q**), and delicate wipers (**R**) are placed on both sides of the binocular. An ice bucket with the ES, the FBS-ES, the intracellular solution syringes, the 60 mm Petri dish (**S**), and the electrode holder (**P**) are also placed on the table. The recording electrodes are pulled using a horizontal puller (**T**). Please click here to view a larger version of this figure.

1. **Construct a retina scooper to isolate the retinae.**
  1. Insert the point (1-4 mm) of a micro dissection needle (entomological needle, 12 mm length, 0.1 mm diameter) between the two vise jaws and flatten it by tapping with a small hammer.
  2. Mount the flattened needle on a micro dissecting needle holder (see the **Table of Materials**).
  3. Using a pair of tweezers, curve the flattened end of the needle to form a hook with a curvature of ~2.5 mm.
2. **Create trituration pipettes for ommatidia separation.**
  1. Place a 1.2 x 0.68 mm (O.D. x I.D.) glass capillary over an open flame and fire polish it to reduce its opening.
  2. Measure the size of the capillary opening under a microscope. Sort the created ommatidial trituration pipettes into seven groups according to the size of their openings (*i.e *0.2-0.5 mm). Store them in separate suitable containers (*e.g.* test tubes).
  3. Fill one small beaker with double-distilled water (DDW) and another small beaker with ethanol 70%. Cover each beaker with a paraffin film sheet.
  4. Punch one small hole in the paraffin film sheet covering the DDW beaker (see **Figure 1A**).
  5. Punch seven small holes in the paraffin film sheet covering the ethanol beaker and number the holes from 0 to 6 (see **Figure 1B**).
  6. Place one ommatidial trituration pipette from each size group in every paraffin film hole, such that the pipette with the largest opening is located in the 0^th^ hole and the pipette with the smallest opening is located in the 6^th^ hole.
  7. Connect a plastic 200 µL pipette tip to a 35 cm long, 1.57 x 1.14-mm (O.D. x I.D.) piece of polyethylene tubing. Connect the other end of the tubing to one of the ommatidial trituration pipettes with the largest opening (*i.e. *0.46 - 0.5 mm), ensuring that the pointed end of the trituration pipette is not facing into the tubing.
3. Prepare whole-cell recording pipettes by pulling patch clamp pipettes from a 1 x 0.58 mm (O.D. x I.D.) borosilicate filament containing glass capillaries. NOTE: The resistance of the pipettes should be 8-15 MΩ when using potassium gluconate-based intracellular solution (IS1). Any suitable patch pipette puller can be used (for examples, see the **Table of Materials**). Fire-polishing is not necessary.
4. Prepare a recording chamber (see **Figure 2**) by fixing a coverslip (see the **Table of Materials**) to the bottom of the bath chamber using melted paraffin or high-vacuum silicone grease. Use any suitable homemade or commercial chamber that allows electrode access and perfusion.
5. Mount the bath on the stage of an inverted microscope. Place the perfusion system tube, suction system, and ground (*i.e. *Ag-AgCl wire/pellet) in the bath (see the **Table of Materials** and **Figure 2**).
6. Place two pairs of fine #5 tweezers (**Figure 1**) on the working surface for use during the retina dissection and ommatidia isolation steps.

### 3. *D. melanogaster* Rearing

1. Raise *D. Melanogaster* flies at a low population density (*i.e. *~20 flies in a 6 oz. bottle) in bottles containing standard cornmeal food at 19-24 °C. NOTE: It is preferable to work on dark-adapted flies. To maintain high sensitivity to light, reduce the diversity and prevent retinal degeneration in mutant flies.
2. Rear the flies in the dark for at least 24 h prior to the experiment. NOTE: The flies used for the experiments should be recently eclosed (<2 h) and still soft, pale, and display the meconium. Ommatidia can also readily be prepared from pupae, though their sensitivity to light is then steeply dependent on age^10^.

### 4. Retina Dissection and Ommatidia Isolation: Option 1

NOTE: Perform all of the following steps under the stereoscopic zoom microscope using an amplification suitable for properly viewing the preparation (See **Figure 1**).

1. Place four drops of ES-0Ca^2+^ and one drop of TS solution on a 60-mm Petri dish that has been turned over.
2. Using rough tweezers, catch a newly eclosed (<2 h after eclosion) fly by its wings or body. From this point on, perform all procedures rapidly and under dim red illumination at 20 ±1 °C.
3. While still grasping the fly with the rough tweezers, use the first pair of fine tweezers to detach the fly head from the body. Submerge the head in the first ES-0Ca^2+^ drop.
4. Dissect the head in half along the sagittal plane using the second pair of fine tweezers. Ensure that, at the end of this step, both eyes are still intact.
5. Transfer one half of the head to the second ES-0Ca^2+^ drop and the other half to the third ES-0Ca^2+^ drop.
6. Using the fine tweezers, remove as much of the tissue surrounding the eye as possible and ensure that no harm is caused to the retina.
7. Firmly grasp the edge of one cornea with the fine tweezers and scoop out the retina using the scooper. NOTE: Upon completion of this step, the cornea will be left empty and intact, separated from an intact retina.
8. Rinse the trituration pipette connected to the tubing with DDW and fill the pipette with a small amount of ES-0Ca^2+^ from the fourth drop. NOTE: This step must be performed every time a new ommatidia-separating pipette is used and removed from the ethanol beaker (the solution filling the pipette should match the solution in which the retina is submerged).
9. Gently aspirate by mouth to draw the isolated retina into the pipette. Use extreme caution not to aspire air bubbles into the pipette.
10. Transfer the isolated retina to the drop of TS. Perform steps 4.6-4.10 on the second eye as well.
11. Wipe away the drops of ES-0Ca^2+^ using delicate wipes, leaving only the drop of TS containing both retinae on the Petri dish. Add six more drops of TS to the top of the Petri dish. Transfer both retinas to one of the other TS drops.
12. Replace the trituration pipette with a pipette of a smaller-diameter opening. Rinse it as described in step 4.8, using TS as the solution to fill the pipette.
13. Rapidly and repeatedly aspirate and expirate both retinae in the solution to begin the separation of isolated ommatidia stripped of pigment cells from the whole retina. NOTE: The isolated ommatidia are visible in the TS drop, and as the isolation process progresses, the TS drop becomes less translucent.
14. Transfer the remaining retinae to the next TS drop. Fill the pipette with the entire former TS drop (containing the isolated ommatidia) and expirate the drop into the bath chamber.
15. Repeat steps 4.12-4.14 to achieve maximal isolated ommatidia. Wait approximately 1 min to allow the isolated ommatidia to sink and bind to the bottom of the bath chamber.
16. Using the perfusion system, start the flow of ES-0Ca^2+^ with 1.5 mM Ca^2+^ into the bath chamber. Ensure that the chamber is completely filled with the solution, from bottom to top, and that the ground is completely submerged in the solution. Continue to wash the bath 4-5x.

### 5. Retina Dissection and Ommatidia Isolation: Option 2

Note: Perform all of the following steps under the stereoscopic zoom microscope, using an amplification suitable for properly viewing the preparation (See **Figure 1**).

1. Prepare a razor blade chip and holder. Break off and mount a small triangular chip of a razor blade using a razor blade holder (**Figure 1**).
2. Prepare a silicone dissection dish/block according to the manufacturer's instructions (see the **Table of Materials**).
3. For the dissection, create a large drop (<0.5 mL) of ES solution on the silicone dissection block. Add two "reservoir" drops (~50 µL each) of TS solution to a 60 mm Petri dish
4. Immobilize a newly eclosed (<2 h post-eclosion) fly in a glass tube on ice and pick it up by its wings using tweezers. From this point on, perform all procedures rapidly and under dim red illumination at 20 ±1 °C.
5. Holding the fly with tweezers, cut off the fly's head using a razor blade chip mounted in a holder. Pick up an insect pin (12 mm long, 0.1 mm in diameter) with the tweezers and pierce the head between the eyes.
6. Briefly submerge the head in 70% ethanol; this prevents air bubbles from forming on the head/eye surface. Pin the head under the ES drop on the silicone dissection dish.
7. Cut off both eyes using the razor blade chip by using a sawing motion along the line of the frontal margin of the eye.
8. Firmly grasp the edge of one cornea with the fine tweezers.
9. Scoop out the retina using the scooper. NOTE: Upon completion of this step, the cornea will be left empty and intact, separated from an intact retina.
10. Without damaging the retina, use the tweezers and scooper to gently remove adhering air sacs and excess brain tissue. NOTE: The preparation of isolated retinae is also useful for Western blot analyses of non-specific retinae proteins^11^, whole-mount histology, and whole-retina imaging. Patch clamp recordings can also be conducted on photoreceptors from the whole retina^12^.
11. Take the trituration pipette with the largest diameter, connect it to the tubing, and backfill the pipette with a small amount of TS from one of the reservoir drops in the Petri dish by gentle suction (*i.e. *by mouth). Perform this step every time a new ommatidia trituration pipette is used.
12. Gently blow the TS over the two retinae by mouth and then draw the isolated retinae into the pipette using gentle suction. Use caution to ensure that no air bubbles enter the pipette.
13. Transfer the isolated retinae to the Petri dish, forming a small drop (~20 µL), and wash them once or twice with TS from one of the reservoir drops.
14. Incubate the retinae in the dark for 20-25 min.
15. Replace the ommatidia trituration pipette with a pipette with a smaller-diameter opening (using fresh TS from one of the reservoir drops as in step 4.6 to backfill the pipette).
16. Rapidly aspirate and expirate both retinae in a small drop (~20 µL) to begin the separation of the isolated ommatidia. NOTE: At the first stage, the surrounding pigmented glia should disintegrate, leaving visible small debris in the solution.
17. After substantial small debris has accumulated, but before many ommatidia have separated, use fresh TS from one of the reservoir drops and transfer the retinae to a new small drop.
18. Select a smaller-diameter trituration pipette, backfill, and continue to triturate. NOTE: As ommatidia now begin to separate, their elongated forms should be clearly visible under the high power of the stereomicroscope. If necessary, keep changing the trituration pipettes to smaller diameters until a good yield of ommatidia is visible
19. Once a reasonable yield of ommatidia are visible and the drop is no longer translucent, fill the pipette with the entire drop containing the isolated ommatidia and expirate the drop gently into the bottom of the bath chamber pre-filled with ES.
20. Wait approximately 1 min to allow the isolated ommatidia to sink and settle on the bottom of the bath chamber.
21. Using the perfusion system, start the flow of ES into the bath chamber. Ensure that the chamber is completely full of the solution, from bottom to top, and that the ground is totally submerged in the solution. Continue to wash the bath 4-5x. NOTE: Thereafter, continuous perfusion is not required, though the bath should be briefly flushed before introducing a new patch pipette.

### 6. Whole-Cell Recording


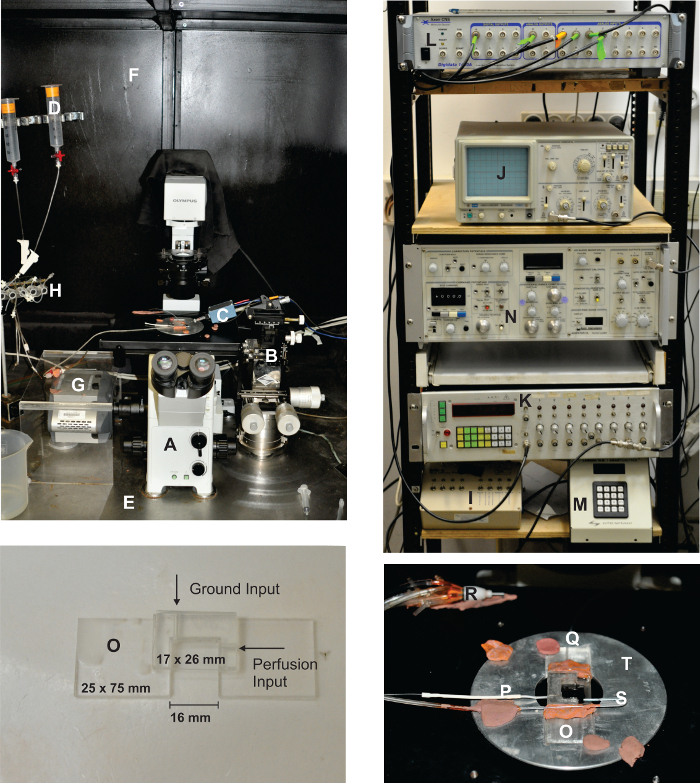
**Figure 2: Overview of the Electrophysiological and Optical Setup. **The setup contains an iron, black-painted Faraday cage (**F**). The front side is covered using a black curtain with copper mesh inside. This configuration enables the preparation to be completely sealed off from any stray light and is suitable for recording dark spontaneous bumps. The inverted fluorescence microscope (**A**) is fixed to an anti-vibration table (**E**). The photoreceptor recording apparatus consists of a homemade acrylic glass bath chamber (**O**) with a perfusion input (**Q**), a suction pipette (**S**), and a silver-silver chloride ground (**P**). The bath chamber is mounted on the microscope stage (**T**) with a homemade adaptor. The recording pipette is connected via silver-silver chloride wire to an acrylic glass holder, which is connected to the amplifier head stage (**C**). The head stage is then mounted on a coarse micromanipulator that is mounted on a fine XYZ mechanical micromanipulator (**B**). The perfusion system (**D**) is composed of a syringe set, while the flow of liquid is controlled by pinch valves (**H**). The rack is electrically connected to the same central ground to which all the equipment inside the Faraday cage is connected and consists of a patch clamp amplifier (**N**), an oscilloscope (**J**), a pulse/function generator (**K**), an A to D converter (**L**), a perfusion controller (**I**), and a filter wheel and shutter controller (**M**). For imaging experiments, a cooled (-110 °C, see the **Table of Materials**) CCD camera is connected via a side port (**G**). Please click here to view a larger version of this figure.

NOTE: During all of the following steps, use only dim red light illumination and ensure that the exposure of the ommatidia to light is minimal (*i.e.* work rapidly and turn off the dissecting light source and chamber red illumination used for viewing the ommatidia when performing tasks that do not demand viewing the ommatidium). In addition, perform all of the following steps according to standard electrophysiological protocol.

1. **Under an inverted microscope (40X objective), carefully inspect all of the ommatidia in the bath and choose a suitable ommatidium for the experiment.**
  1. Ensure that the outer membrane of the ommatidium is smooth and intact, that the long axis is approximately at a right angle relative to the electrode approach direction (as seen in **Figure 3A**), and that the distal section of the ommatidium is not surrounded by any excess tissue. Place the chosen ommatidium at the optical axis of the objective lens (in the center of the field of vision) to ensure uniform illumination.
2. Fill a patch pipette with intracellular solution (IS1 or IS2). NOTE: To measure the intensity response and bump analysis, use IS1. To measure the reversal potential of the light-sensitive channels, use IS2.
3. Mount the patch pipette onto the electrode holder.
4. Blow into the pipette by mouth, through the tube connected to the electrode holder, causing it to fill with positive pressure. Close the tube valve to maintain the pressure.
5. Using the micromanipulator, insert the electrode into the bath chamber.
6. Guide the electrode close to the distal section of the ommatidium, such that there is no contact between the electrode and the ommatidium, until a small dimple (due to the positive pressure in the patch pipette) can be observed in the ommatidium.
7. Open the recording software (see the **Table of Materials**). Open the "membrane test module" to apply continuous square voltage pulses of 2 mV at a rate of 100 Hz.
8. Set the junction potential to "zero," by adjusting the appropriate knob in the patch clamp amplifier to set the base of the square pulse to "zero" current NOTE: The electrophysiological setup includes a head stage (*i.e. *first-stage amplification) connected to an amplifier (*i.e. *second-stage amplification). The amplified analogue signal is converted to a digital signal using the A/D converter, which is controlled by software installed on a PC computer.
9. Release the positive pressure in the pipette by opening the valve of the tube connected to the electrode holder. Gently create negative pressure in the pipette by sucking out of the tube, leading to the association of the pipette to the cell membrane. Close the tube valve to maintain the pressure.
10. Ensure that the electrode resistance viewed on the computer screen is elevated to 100 - 150 MΩ. Release the negative pressure in the pipette by manually opening the valve of the tube connected to the electrode holder.
11. Ensure that the electrode resistance is elevated to at least 1-2 GΩ. NOTE: At this point, a seal has been formed between the electrode and the photoreceptor.
12. Offset the capacitive currents of the pipette by adjusting the appropriate knob in the patch clamp amplifier.
13. Create rapid, short, and forceful bouts of negative pressure in the electrode, sucking by mouth out of the tube connected to the electrode holder to "break" into the photoreceptor membrane and create a whole-cell configuration. Alternatively, use the "Zap button" to apply short, rectangular electrical pulses, starting with a duration of "0.1 ms," or apply a combination of both methods. NOTE: The generation of the whole-cell configuration is revealed by a sudden increase in pipette capacitance (typically ~60 pF for a wildtype R1-6 photoreceptor; a capacitance of only ~20 pF indicates a recording from an R7 photoreceptor; a capacitance above ~90 pF indicates a recording from two photoreceptors).
14. Set the holding potential of the photoreceptor to the required voltage (usually, -70 mV), manually using the appropriate knob in the patch clamp amplifier. NOTE: It is possible to perform this step after a seal has been obtained (step 6.11) and before the whole-cell configuration has been achieved.
15. Offset the capacitive currents and series resistance (a measured series resistance value greater than 25 MΩ indicates that the electrode pipette has been clogged) and, if required (*i.e. *for larger currents), apply series resistance compensation using the appropriate knobs in the patch clamp amplifier.
16. Close the black front curtain of the Faraday cage to obtain maximal darkness and electrical isolation.
17. Begin the recording process using the software and administer light stimuli and/or pharmacological substances according to the desired experimental procedure.

### 7. Simultaneous Whole-Cell Recordings and Ca^2+^ Imaging

1. For genetically encoded Ca^2+^ indicators, isolate the ommatidia as described above using *D. melanogaster* flies expressing GCaMP6f^13^. Use a CCD camera (see the **Table of Materials**) for the fluorescence measurement and ensure that the microscope is equipped with proper excitation and emission filters and a dichroic mirror (see the **Table of Materials**)^4^.
2. For the use of exogenous Ca^2+^ indicator (**Figure 4**; see the **Table of Materials**), isolate the ommatidia as described above. In addition, ensure that the pipette solution includes a 20-100 µM calcium indicator.
3. Use the imaging software (see the **Table of Materials**) to acquire images at a rate of 40 Hz. Perform image acquisition during a 10 s dark period followed by an intense 2 s light stimulation.
4. Use the imaging software and define a Region of Interest (ROI). Measure the florescence intensity in the ROI. Average the dark fluorescence (F_D_) and subtract it from the fluorescence recordings during the light (F_L_) stimulation (F_L_-F_D_). Normalize these measurements according to the fluorescence intensity at the beginning of the light stimulation (F_L_^0^).

## Representative Results

The described method has enabled the accurate recording of the fundamental unitary currents that generate spontaneous and light-evoked quantum bumps, which sum to produce the macroscopic response to light, under defined conditions. It also allowed the comparison between wildtype and mutant flies that have defects in critical signaling molecules (**Figures 3 **and** 5**)^14^,^15^,^16^,^17^,^18^. In addition, the ability to measure reversal potential under bi-ionic conditions revealed fundamental biophysical properties of the TRP and TRP-like (TRPL) channels^18^,^19^. It also enabled the measurement of the effects of amino acid substitutions in the pore region of TRP that modified its Ca^2+^ permeability^20^.

The light response obtained by patch clamp whole-cell recordings depends linearly on light intensity for at least 4 orders of magnitude. This could not be resolved by using ERG and intracellular recording methods. Accordingly, a series of responses to brief flashes of increasing intensity and a plot of the intensity response function revealed a strict linearity of the flash response with increasing light intensity. The strict linearity holds up to at least several hundred pA, but it is debatable whether thereafter it is linearity or clamp control that breaks down (**Figure 6**). These results suggest that the macroscopic responses to light are a linear summation of the unitary responses to light (*i.e.* quantum bumps).

It has been well established using voltage recordings that dim light stimulation induces discrete voltage fluctuations (*i.e. *quantum bumps) in most invertebrate species. The *D. melanogaster* quantum bumps result from the concerted opening of ~15 TRP channels and ~2 TRPL channels at the peak of the bump^18^. Each bump is generated by the absorption of a single photon, while the macroscopic response to more intense lights is the summation of these elementary responses^14^,^21^. The bumps vary significantly in latency, time course, and amplitude, even when the stimulus conditions are identical. Bump generation is a stochastic process described by Poisson statistics, whereby each effectively absorbed photon elicits only one bump. The single-photon-single-bump relationship requires that each step in the cascade includes not only an efficient "turn-on" mechanism, but also an equally effective "turn-off" mechanism. The functional advantage is the production of a very sensitive photon counter with a fast transient response very well suited to both the sensitivity and the temporal resolution required by the visual system. The requirement for an efficient turn-off mechanism is revealed when either the active photopigment (*i.e. *metarhodopsin, M) or its target, the G_q_α, fails to inactivate and leads to the continuous production of bumps long after light is turned off (**Figure 3**)^15^,^22^,^23^,^24^ .

The bump represents the cooperative activity of the TRP/TRPL channels in a microvillus. As such, any hypothesis of channel activation should also explain cooperative channel activation. Recently, Hardie and colleagues have demonstrated that light evokes rapid contractions of the photoreceptors, suggesting that the light-sensitive channels (TRP/TRPL) may be mechanically gated^25^. This mechanical activation, together with the observed protons released by PLC-mediated PIP_2_ hydrolysis, promote the opening of the TRP/TRPL channels and explain the cooperative nature of bump production^26^. Currently, *D. melanogaster* photoreceptors are one of the few systems in which phosphoinositide signaling and TRP channels can be studied *in vivo*, thus making *D. melanogaster* phototransduction and the methodology developed to study this mechanism a highly valuable model system.


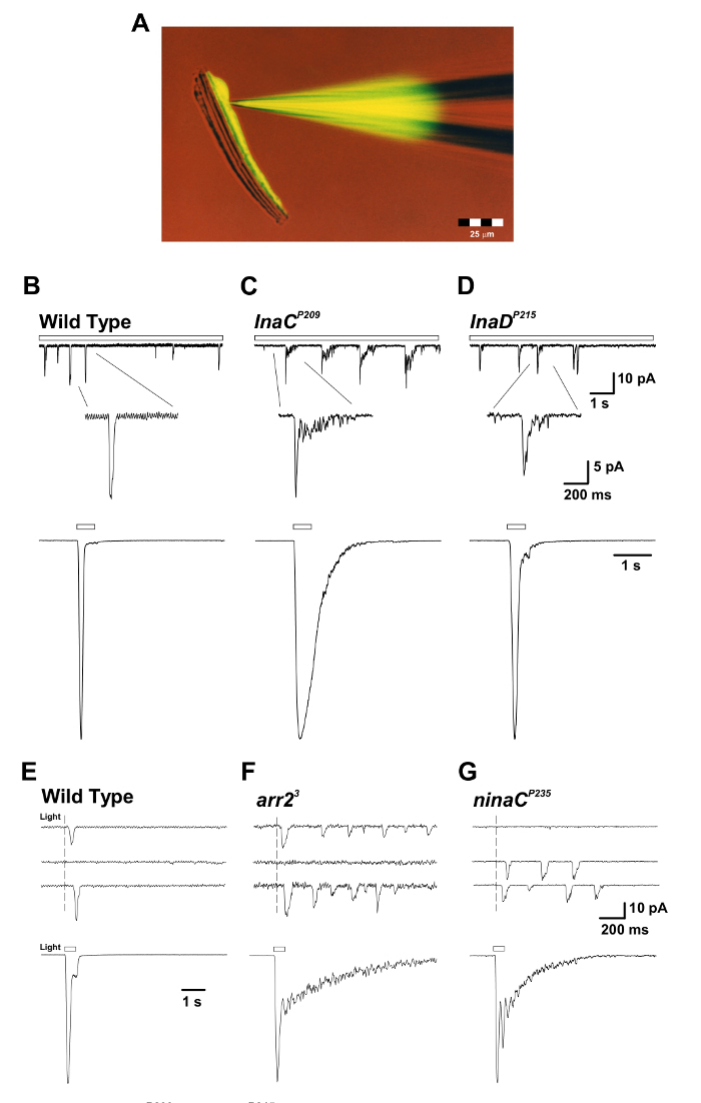
**Figure 3: The inaC^P209^ and inaD^P215 ^Mutants Reveal Slow Response Termination of the Macroscopic Response to Light and of the Single Quantum Bumps.****(**A**)The isolated ommatidium preparation with a patch pipette filled with fluorescent Lucifer Yellow CH dye (excitation: 430 nm; emission: 540 nm) is presented during a whole-cell recording. Note that the fluorescent dye diffused and labeled a single photoreceptor cell body and that the photoreceptor cell bodies are detached from their elongated axons but still maintain viability. This preparation is suitable for simultaneous whole-cell recordings and imaging experiments. (**B-D**) Upper panels: Whole-cell voltage clamped quantum bump responses to continuous dim light (open bar) in WT, *inaC^P209^*, and* inaD^P215^*mutant flies. A slow termination of the bumps is observed in* inaC^P209 ^*and* inaD^P215^*mutants relative to WT flies. The inset below displays the magnified shape of single bumps. Bottom panels: Normalized whole-cell recorded macroscopic responses to a 500-ms light pulse (1.5 x 10^5^ photons per s) of the above wildtype and mutant flies. **(E-G) **Upper panels: Whole-cell voltage clamped quantum bump responses to a brief (1 ms), dim light eliciting single-photon responses in wildtype, *arr2^3^*, and* ninaC^P235 ^*mutant flies. Note the train of bumps observed in *arr2^3^* and *ninaC^P235 ^*mutant flies in response to a single photon absorption. Bottom panels: Whole-cell voltage clamped normalized responses to a 500 ms light pulse (1.5 x 10^4^ photons/s) in the corresponding mutants. Note the slow termination of the macroscopic responses observed in *arr2^3^*****and *ninaC^P235 ^*mutant flies relative to WT. Please click here to view a larger version of this figure.


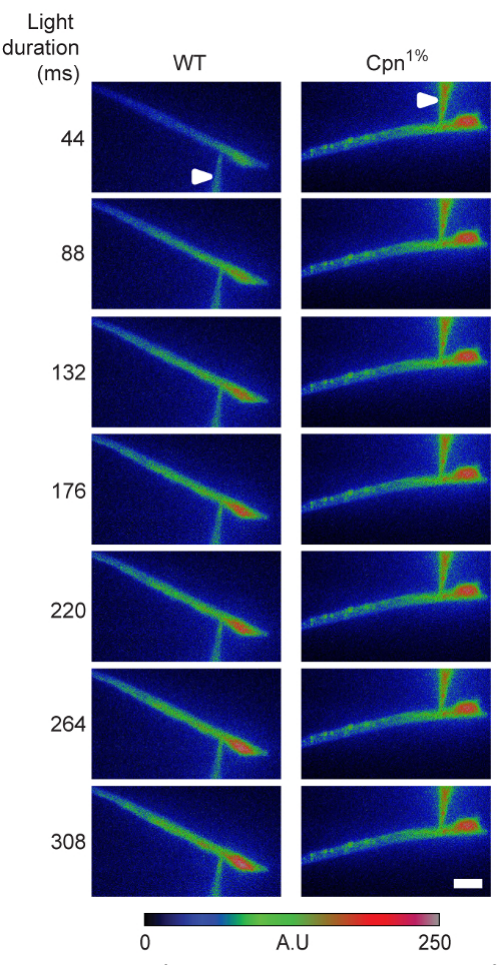
**Figure 4: Cellular Ca^2+^ Dynamics Following Signal-induced Ca^2+^ Influx is Affected by Calphotin.  **A time series of photoreceptor images of wildtype and Cpn^1%^ flies showing the fluorescence of the Ca^2+^ indicator during light stimulation. Raw intensity images are plotted using false-color coding (bar = 10 µm; arrowheads indicate the pipette). Figure reprinted with permission from Weiss *et al.*^4^. Please click here to view a larger version of this figure.


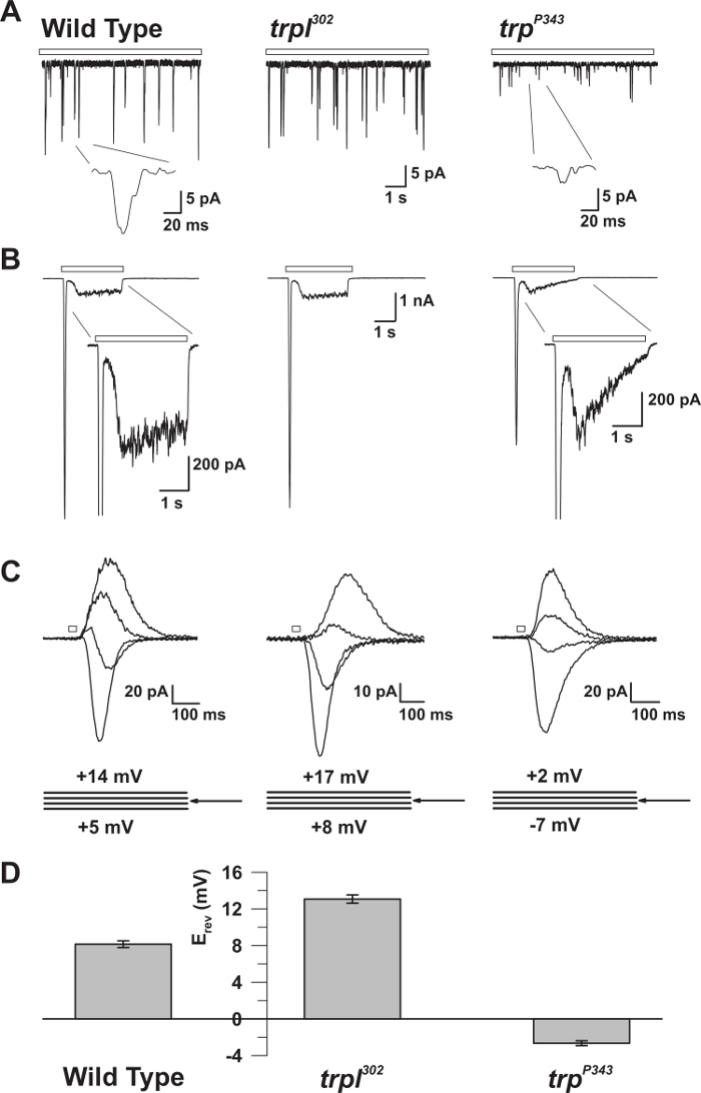
**Figure 5: The Electrophysiological Properties of WT, trp, and trpl Mutants.********(**A**) Whole-cell voltage clamp recordings of quantum bumps in response to continuous dim light (open bar) in WT, trpl^302^, and trp^P343^ null mutant flies. Highly reduced amplitudes of *trp*^P343^ bumps are observed. Inset: Magnified single quantum bumps of wildtype and *trp^P343^* null mutant flies are shown. (**B**) Whole-cell voltage clamp recordings in response to a 3 s light pulse of wildtype and the corresponding mutants. The transient steady-state response of the *trp^P343 ^*mutant is observed. Inset: Magnified light responses of WT and *trp^P343^*mutant are shown. (**C**) A family of superimposed light-induced currents of the above fly strains, elicited in response to a 20 ms light pulse, at voltage steps of 3 mV, measured around the reversal potential (E_rev_). (**D**)****A histogram plotting the mean E_rev_ of wildtype and the various mutants. The error bars are the S.E.M. The reversal potential (E_rev_) of WT is between the positive E_rev_ of *trpl^302^*, which expresses only TRP, and the E_rev _of the *trp^P343^* null mutant, which expresses only TRPL. Please click here to view a larger version of this figure.


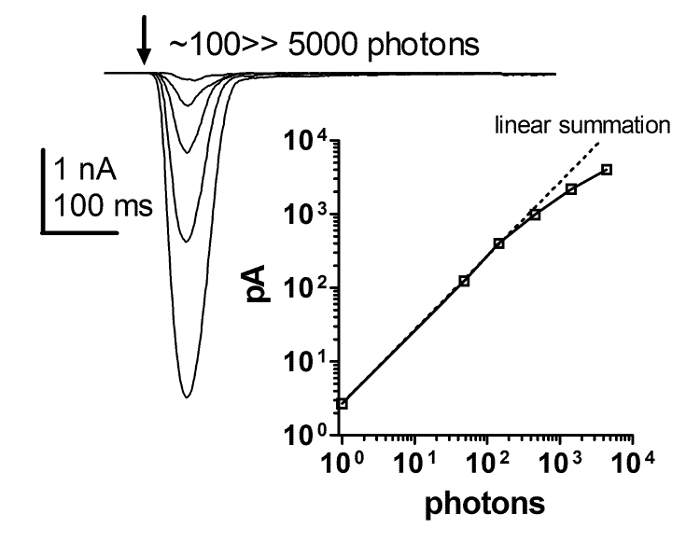
**Figure 6: The Flash Response is Strictly Linear with Increasing Light Intensity.** A series of current responses to brief flashes of increasing light intensity and a plot of the dependence of the peak amplitude of the light response on the increasing intensity of brief light flashes. This relationship reveals a strict linearity between the flash response and increasing light intensity. This strict linearity holds up to at least several hundred pA, with light intensity spanning over 4 orders of magnitude, while it is debatable whether it is linearity or the clamp control that breaks down thereafter. Please click here to view a larger version of this figure.

**Table:** 

pH	7.15 (adjust with NaOH)
**Reagent**	**Concentration (mM)**
NaCl	120
KCl	5
MgCl_2_	4
TES	10
Proline	25
Alanine	5
	Store at -20 °C.
Note: This solution is nominally Ca^2+^ free but has no Ca^2+^ buffers added and hence will have approximately 5 - 10 µM trace Ca^2+^. Extracellular solution (ES) = ES-0Ca^2+^ with 1.5 mM CaCl_2_, made by adding CaCl_2_ from a 0.5 or 1 M stock solution to ES-0Ca^2+^.

**Table 1: Ca^+2^-free Extracellular Solution (ES). **Chemical description and the specific quantities required to produce Ca^+2^-free ES.

**Table:** 

**Reagent**	**Amount**
FBS	15 mL
sucrose	1.5 g
Divide into 150 µL aliquots in 1.5 mL vials and store at -20°C.
Trituration solution (TS)	Fill 1 vial of 150 mL of the stock solution with 1,350 mL ES or ES-0Ca^2+^, to match the solution used during the dissection.

**Table 2: Fetal Bovine Serum (FBS) + Sucrose - Stock Solution. **Chemical description and the specific quantities required to producing fetal bovine serum (FBS) + sucrose - stock solution.

**Table:** 

pH	7.15 (adjust with KOH)
**Reagent**	**Concentration (mM)**
Potassium gluconate (Kglu)	140
MgCl_2_	2
TES	10
ATP magnesium salt (MgATP)	4
GTP sodium salt (Na_2_GTP)	0.4
β-Nicotinamide adenine dinucleotide hydrate (NAD)	1
	Store at -20 °C.

**Table 3: Intracellular Solution (IS1). **Chemical description and the specific quantities required to produce IS1, which is mostly used for intensity response and quantum bump measurements.

**Table:** 

pH	7.15 (adjust with CsOH)
**Reagent**	**Concentration (mM)**
CsCl	120
MgCl_2_	2
TES	10
ATP magnesium salt (MgATP)	4
GTP sodium salt (Na_2_GTP)	0.4
β-Nicotinamide adenine dinucleotide hydrate (NAD)	1
Tetra-ethyl-ammonium chloride (TAE)	15
	Store at -20 °C.

**Table 4: Intracellular Solution (IS2). **Chemical description and the specific quantities required to produce Intracellular Solution IS2, which is mostly used for reversal potential measurements of the light-induced current.

## Discussion

The application of whole-cell recordings to *D. melanogaster* photoreceptors allowed for the discovery and the functional elucidation of novel signaling proteins, such as TRP channels^27^,^28^,^29^ and INAD^30^,^31^,^32^ scaffold protein. Ever since the initial introduction of this technique, it enabled the resolution of long-term basic questions regarding the ionic mechanism and voltage dependence of the light response. This occurred because of the conferred ability to accurately control the membrane voltage and extracellular and intracellular ionic composition^19^,^28^.

A major obstacle of the patch clamping technique in *D. melanogaster* has been the fragility of the isolated ommatidia preparation. Detailed studies have revealed that the integrity of the phototransduction machinery critically depends on the continuous supply of ATP, especially during light exposure, which leads to a large consumption of ATP. Unfortunately, the mechanical striping of the pigment (*i.e. *glia) cells, which is required to reach the photoreceptor membrane with the patch pipette, eliminates the main source of metabolites necessary for ATP production^33^. Application of exogenous ATP into the recording pipette only partially fulfills the requirement for large quantities of ATP. A short supply of ATP leads to spontaneous activation of the TRP channels and to the dissociation of the phototransduction machinery from the light-activated channels, causing a large increase in cellular Ca^2+^ and the abolishment of the normal response to light^34^,^35^. This sequence of events is not due to damage of the photoreceptors by the dissection procedure, but rather to the cellular depletion of ATP. To prevent this sequence of events from occurring and to maintain normal light responses, the photoreceptors should not be exposed to intense lights, which consume large quantities of ATP. Also, NAD must be included in the recording pipette, presumably to facilitate ATP production in the mitochondria^18^,^36^. For measurements of spontaneous and quantum bumps, the above difficulty is minimal because only dim lights are used. In practice, a stable whole-cell recording can be maintained for ~20-25 min, although there is a tendency for response kinetics to slow down over this period. A single preparation of dissociated ommatidia may remain viable for up to 2 h.

An additional shortcoming of the isolated ommatidia preparation is the inaccessibility of the microvilli, which translates to the inaccessibility of the TRP and TRPL channels to the recording pipette, preventing single-channel recordings. Using a method they developed, Bacigalupo and colleagues succeeded at directly recording single-channel activity from the rhabdomere^37^. However, this channel activity differs from that of TRPL channels heterologously expressed in tissue culture cells^38^ and from TRP channel activity derived from shot noise analysis obtained from isolated ommatidia^34^. Presumably, the dissection procedure greatly damaged the photoreceptor cells when using this method.

## Disclosures

The authors have nothing to disclose.
